# Indirect calibration between clinical observers - application to the New York Heart Association functional classification system

**DOI:** 10.1186/1756-0500-4-276

**Published:** 2011-08-03

**Authors:** Milton Severo, Rita Gaio, Patrícia Lourenço, Margarida Alvelos, Paulo Bettencourt, Ana Azevedo

**Affiliations:** 1 Department of Clinical Epidemiology, Predictive Medicine and Public Health, University of Porto Medical School, Porto, Portugal; 2 Institute of Public Health of the University of Porto, Porto, Portugal; 3 Department of Pure Mathematics, University of Porto Science School, Portugal; 4 Heart Failure Clinic, Department of Internal Medicine, Hospital S. João, Porto, Portugal

**Keywords:** dyspnea, physical exertion, questionnaires, New York Heart Association, calibration, reliability, equating

## Abstract

**Background:**

Previous studies showed an inter-observer agreement for the NYHA classification of approximately 55%. The aim of this study was to calibrate the New York Heart Association (NYHA) classification system between observers, increasing its reliability.

**Results:**

Among 1136 community-dwellers in Porto, Portugal, aged ≥ 45 years, 265 reporting breathlessness answered a 4-item questionnaire to characterize symptom severity. The questionnaire was administered by 7 physicians who also classified the subject's functional capacity according to NYHA. Each subject was assessed by one physician. We calibrated NYHA classifications by the concurrent method, using 1-parameter logistic graded response model. Discrepancies between observers were assessed by differences in ability thresholds between NYHA classes I-II and II-III. The ability estimated by the model was used to predict the NYHA classification for each observer.

Estimates of the first and second thresholds for each observer ranged from -1.92 to 0.46 and from 1.42 to 2.30, respectively. The agreement between estimated ability and the observers' NYHA classification was 88% (kappa = 0.61).

**Conclusions:**

The study objectively indicates the main reason why several studies have reported low inter-observer is the existence of discrepant thresholds between observers in the definition of NYHA classes. The concurrent method can be used to minimize the reliability problem of NYHA classification.

## Background

The New York Heart Association (NYHA) functional classification was originally conceptualized and described in 1928 and most recently updated in 1994 as a method of assessing functional disability induced by cardiac diseases in patients encountered in clinical practice [1]. The NYHA system was designed for clinical assessment of patients by physicians in 4 classes (I, II, III or IV) on the basis of the patient's limitations in physical activities caused by cardiac symptoms. The NYHA classification is derived largely by inference from history and/or observation of the patient in certain physical activities, and occasionally by direct or indirect measurement of cardiac function in response to standardized exercises. There was an attempt to increase the objectivity of the NYHA classification by adding an objective assessment, based on measurements such as electrocardiogram, stress test, X-ray and echocardiogram. Despite this attempt, the NYHA classification remains essentially subjective [2]. The class a clinician decides to assign a patient to depends on the clinician's interpretation of what is "ordinary" physical activity, "slight" and "marked" limitations. This results in a high inter-observer variability. Previous studies showed an inter-observer agreement for the NYHA classification of approximately 55% [3,4]. Consequently the use of NYHA classification as an outcome measure in clinical research is rather poor. However this classification system has been widely used in clinical epidemiology studies as an inclusion criterion and also as an outcome measure [2]. It is also used in routine clinical practice.

The aim of this study was to calibrate the NYHA classification system between different observers, aspiring to increase its reliability, by quantifying the discrepancy in thresholds in functional capacity that lead an observer to assign a NYHA class to a patient.

## Methods

Participants were selected within the first follow-up of a cohort, representative at baseline of the non-institutionalized adult population of Porto, Portugal - the EPIPorto cohort study. At baseline, households were selected by random digit dialling [5]. After the identification of a household, permanent residents were characterized according to age and gender, and one individual aged 18 years or older was randomly selected and invited to visit our department for an interview and physical examination. If there was a refusal, replacement was not allowed within the same household.

Trained interviewers collected information, using a standard protocol that comprised questions on social, demographic, clinical and behavioural characteristics. At baseline, 2485 participants were recruited. Between October 2006 and July 2008, all participants aged ≥ 45 years were eligible to a systematic evaluation, at our department, of measures of cardiac structure and function, which included a cardiovascular clinical history and physical examination, and a transthoracic echocardiogram.

Among 2048 eligible to this study, 134 (6.5%) had died, 198 (9.7%) refused to be re-evaluated and 580 (28.3%) were lost to follow up (unreachable by telephone or post). Therefore 1136 (55.4%) individuals aged ≥ 45 years were assessed by 8 physicians experienced in the management of heart failure patients.

At the standardized clinical interview applied by these physicians, subjects who reported to have breathlessness (n = 265; 23.3%) were presented to a 4-item questionnaire on functional capacity to characterize the severity of symptoms: 1) whether breathlessness is felt when walking on steep plane, horizontal plane or at rest; 2) distance walked until perception of breathlessness; 3) sets of stairs (10-15 steps) climbed until perception of breathlessness; 4) whether mild, moderate or intense efforts are necessary to elicit breathlessness. These will hereafter be referred to as "anchor items".

The same physician administered the questionnaire and classified the subject's functional capacity using the NYHA classification. This classification, defined by each physician for each subject, will hereafter be referred to as "target items". The assessment of the NYHA classification was carried out after the administration of the 4 anchor items. NYHA class IV was aggregated to class NYHA III because only one individual was classified in NYHA IV.

The Medical Outcomes Study Short Form-36 (SF36) was used to assess health-related quality of life [6]. The scale had been previously translated and the adapted Portuguese version was validated [7]; each sub-domain of the SF-36 is scored from 0 to 100, with increasing values representing better health. Participants completed a physical activity questionnaire designed to estimate usual individual daily energy expenditure, focused on the activity in the past year. Time spent in a variety of activities per day, including work, transport to and from work, household chores, sports, sedentary leisure time and sleep, was self-reported and activity intensity categorized as very light, light, moderate and heavy with a corresponding average of 1.5, 2.5, 5.0 and 7.0 METs respectively, where one MET is equal to the energy expended at the basal metabolic rate or at rest [8]. A severity scale was applied to measure fatigue [9], with increasing values representing higher severity.

The local ethics committee (Hospital São João) approved the study and participants provided written informed consent.

### Statistical analysis

Different correlation coefficients were used to evaluate the magnitude of the association between anchor items and the target items (NYHA classifications): correlations between two (artificial) ordinal variables were evaluated through polychoric correlations, and between interval and (artificial) ordinal variables through polyserial correlations.

Exploratory factor analyses (weighted least square) on the 4 ordinal anchor items combined with each target item was used to evaluate homogeneity (i.e., to confirm there was a single latent variable) of the items and the Cronbach's alpha was used to measure the reliability [10]. The global goodness of fit of the underlying structure with 1 factor was evaluated using the comparative fit index (CFI) recommended when N < 250 [11].

The convergent and divergent validity of the 4 anchor items was assessed through the correlation between the questionnaire's raw score and the 4 physical dimensions of the health-related quality of life scale SF36 (physical function, role physical, bodily pain and general health perception), a scale for fatigue and daily physical activity. The raw score was estimated by the sum of all anchor items.

### Calibration

Each set of individuals assessed by each physician was considered as a group. Calibration of NYHA classification across different groups was performed by the concurrent method. Concurrent calibration involves estimating item and ability parameters in all groups simultaneously, i.e., by combining data from these distinct groups. Items not taken by one of the groups are treated as either not reached or missing [12]. Given the ordinal nature of the items, this is a particular use of the 1-dimensional logistic graded response model (GRM) from item response theory (IRT). Fit of the model was based on approximate marginal Maximum Likelihood. The four patient items were used as anchor items and the 7 obtained NYHA classifications as target items (observer 3 NYHA classification was eliminated for the GRM and dyspnea item was aggregated in two classes 0 vs. 1 and 2 because of the small sample size).

Exploratory factor analysis (EFA) supported that only 1 dimension was reflected in the ordinal items. Thus, 1-dimensional logistic graded response models (GRM) from item response theory (IRT) were used [13]. These models assume that the performance of an individual on the items is explained by only one (standard normal) variable, commonly called "ability". "Ability" is the term that denotes the unobserved hypothetical variable (a latent trait) subjacent to graded response models. In our study, ability refers to the functional capacity of the subject that we are trying to characterize. Higher ability values represent worse functional capacity (more severe symptoms). In the graded response models, each item is described by a set of curves, item operation characteristic curves (IOCC). The item operation characteristic curves for category k represent the probability of endorsing categories higher than k conditional on subject's ability.

The item operation characteristic curves of an item are characterized by several parameters: the slope (discrimination), which is the same for all categories, and the thresholds (difficulty), which are as many as the number of categories minus one. For example, one item with 3 categories has 3 category characteristic curves, one slope and two thresholds: t_1 _to define I versus II-IV and t_2 _to define I-II versus III-IV.

The threshold parameter between two categories represents the ability value at which the probability of indicating the highest of these two or higher is 50%. So, the threshold parameters are expressed in the same scale as the ability. The slope parameter indicates how well an item is able to discriminate individuals with ability values near the respective threshold. The slope parameter may also be interpreted as describing how an item may be related to the ability. The steeper the slope the higher is the item discrimination. We fitted a 1-parameter logistic (1-PL) GRM assuming a unique slope (discrimination parameter) for all items.

### Quality of the calibration

The thresholds estimated for each observer were used as ability cut-off points to predict the observed NYHA classifications, this procedure permitted to assess the ability fit with the target items and the agreement between observers.

In the first case NYHA predictions were sample-specific, i.e., the NYHA predictions were estimated separately for each sample assessed by each of the observers and compared with the observed NYHA classifications.

In the second case NYHA prediction were not sample-specific, i.e., all individuals were classify using the thresholds estimated for each observer regardless of the observer that assessed each individual and compare with each other.

The agreement was assessed with both the absolute agreement and the Cohen's weighted kappa coefficient. Guidelines for interpreting kappa statistics suggest that values between 0.81-1.00 indicate almost perfect agreement, 0.61-0.80 substantial agreement, 0.41-0.60 moderate agreement, 0.21-0.40 fair agreement, and values less than 0.21 are poor or slight agreement [14].

Statistical analyses were performed using the software R 2.12.1 [15], and specifically, the ltm [16] and plink packages and the Mplus software [17].

## Results

The number of individuals assessed by each observer ranged from 10 (3.7%) to 80 (30.4%). The participants were similar by each observer in terms of sex, education and clinical history, systolic blood pressure but showed significant differences in age, body mass index and diastolic blood pressure (Table 1). The NYHA classification showed significant differences by observer, with the prevalence of class I, II and III/IV ranging from 9.3 to 58.8%, 29.2 to 83.3% and 4.8% to 20.0%, respectively. Missing data for the anchor items was equal to or less than 3.0% for all items with the exception of the item 2, 12%. The distribution of NYHA classification in the sample was 85 (33.3%), 147 (57.6%) and 23 (9.0%) for class I, II and III-IV, respectively.

**Table 1:** Characteristics of the study sample by observers

Observer	total	1	2	3	4	5	6	7	8	P
	**N (%)**	**N (%)**	**N (%)**	**N (%)**	**N (%)**	**N (%)**	**N (%)**	**N (%)**	**N (%)**	

Total	265 (100)	21 (7.7)	61 (22.5)	10 (3.7)	80 (30.4)	21 (7.7)	18 (6.6)	24 (8.9)	28 (10.3)	< 0.001
Men	68 (25.7)	7 (33.3)	21 (34.4)	5 (50.0)	14 (17.5)	5 (23.8)	4 (22.2)	7 (29.2)	5 (17.9)	0.180
History of myocardial infarction	19 (7.2)	1 (4.8)	4 (6.6)	1 (10.0)	7 (8.7)	1 (5.0)	0 (0.0)	3 (12.5)	2 (7.1)	0.870
History of angina	32 (12.1)	3 (14.3)	12 (19.2)	1 (10.0)	8 (10.0)	1 (4.8)	1 (5.9)	3 (12.5)	3 (10.7)	0.606
History of heart failure	43 (16.3)	3 (14.3)	13 (21.3)	1 (10.0)	15 (18.8)	1 (4.8)	1 (5.9)	5 (20.8)	4 (14.3)	0.582
Left ventricular systolic dysfunction	**18 (7.1)**	**1 (4.8)**	**6 (10.2)**	**0 (0.0)**	**5 (6.6)**	**0 (0.0)**	**0 (0.0)**	**3 (13.0)**	**3 (13.0)**	**0.571**

NYHA classification										

I	85 (33.3)	10 (47.6)	26 (43.3)	3 (30.0)	7 (9.3)	10 (58.8)	2 (11.1)	15 (62.5)	12 (42.9)	< 0.001
II	147 (57.6)	10 (47.6)	29 (48.3)	5 (50.0)	61 (81.3)	5 (29.4)	15 (83.3)	7 (29.2)	14 (50.0)	
III and IV	23 (9.0)	1 (4.8)	5 (8.3)	2 (20.0)	7 (9.3)	2 (11.8)	1 (5.6)	2 (8.3)	2 (7.1)	

	**Mean** **(SD)**	**Mean (SD)**	**Mean (SD)**	**Mean (SD)**	**Mean (SD)**	**Mean (SD)**	**Mean (SD)**	**Mean (SD)**	**Mean (SD)**	

Age (years)	65.8 (9.7)	67.5 (9.1)	67.6 (10.3)	66.8 (10.5)	65.6 (9.1)	68.6 (7.9)	62.1 (7.9)	60.0 (8.7)	67.1 (10.3)	0.021
Systolic blood pressure (mmHg)	136 (21)	128 (19)	137 (23)	142 (15)	135 (21)	142 (22)	136 (23)	138 (20)	137 (21)	0.574
Diastolic blood pressure (mmHg)	80 (12)	81 (10)	79 (13)	84 (13)	77 (10)	77 (12)	88 (15)	82 (10)	81 (14)	0.025
Body mass index (kg/m^2^)	30 (5.5)	31.4 (7.1)	28.7 (4.5)	28.8 (6.3)	29.4 (4.7)	27.9 (4.6)	33.0 (6.1)	32.9 (5.9)	31.2 (6.4)	0.001

	**Med (IQR)**	**Med (IQR)**	**Med (IQR)**	**Med (IQR)**	**Med (IQR)**	**Med (IQR)**	**Med (IQR)**	**Med (IQR)**	**Med (IQR)**	

Education (years)	4 (5)	4 (4)	4 (3)	7 (7)	4 (5)	4 (7)	5 (3)	4 (6)	4 (0)	0.368

SD: standard deviation

Med: median

IQR: interquartile range.

### Homogeneity

The polychoric correlations between each item and the NYHA classification of all observers were positive and statistically significant (Table 2).

**Table 2:** Score of each anchor item, the distribution of the items and the polychoric correlation of each item with NYHA classification

	Raw Score	N (%)	r^+^
**Total**		**N = 265**	

Do you usually have breathlessness or difficulty breathing? ("dyspnea")		N = 263 (99.2)	

Yes, when walking on steep plane	0	185 (70.3)	0.57
Yes, when walking on the horizontal plane	1	68 (25.9)	
Yes, even at rest	2	10 (3.8)	

If yes, how long can you walk before you have to stop? ("distance")*		N = 232 (87.5)	

0-100 metres	2	97 (41.8)	0.33
101-500 metres	1	86 (37.1)	
501-2500 metres	0	49 (21.1)	

If yes, after how many sets of stairs (10-15 steps) do you have to stop? ("stairs")*		N = 258 (97.3)	

1 set	2	93 (36.0)	0.66
2 sets	1	75 (29.1)	
3 or more sets	0	90 (34.9)	

If yes, in your view, what level of effort induces breathlessness? ("effort")		N = 257 (97.0)	

Great efforts	0	105 (40.9)	0.67
Average efforts	1	89 (34.6)	
Small efforts	2	63 (24.5)	

		Median (IQR)	

Raw score (0-8)		4 (2-6)	0.62

* inverse order for the final score

+ polychoric correlation between each item and NYHA classification of all observers

IQR: interquartile range.

Exploratory factor analysis conducted separately for the 7 observers combined with the 4 anchor items revealed a first factor that accounted more than 70% of the variance, and the first eigenvalue was 3.4 times larger than the second eigenvalue. The fit index met the criteria to support the 1 factor structure, the CFI ranged from 0.934 to 1.00 with the exception of observer 1 than obtained a value of 0.687. The Cronbach's alpha ranged from 0.607 and 0.839 for the 4 common items combined with each observer NYHA classification (Table 3).

**Table 3:** Exploratory factor analysis and internal consistency conducted separately for the 7 observers NYHA classification (target items) and combined with the 4 anchor items

Item	Eigenvalue1	Eigenvalue2	CFI^1^	Alpha Cronbach
Observer 1	3.261	1.856	0.687	0.799
Observer 2	3.383	0.929	0.988	0.778
Observer 3	---	---	---	---
Observer 4	3.754	0.803	0.992	0.790
Observer 5	3.972	0.864	0.987	0.839
Observer 6	3.056	1.406	0.934	0.607
Observer 7	3.363	1.178	0.984	0.732
Observer 8	3.368	0.863	1.000	0.791

^1^Comparative Fit Index.

### Validity of the anchor items

The raw score on the 4-item questionnaire showed a positive correlation with NYHA classification (Table 2). The raw score showed a moderate negative correlation with the 4 physical dimensions of SF36, a positive correlation with the severity of fatigue and no association with total physical activity. The correlations between NYHA classification and SF36, fatigue and physical activity were similar to the ones obtained with the raw score of the questionnaire (Table 4).

**Table 4:** Correlation between the raw score (sum of 4 items) and NYHA with fatigue scale, the daily physical activity, the 4 physical sub-dimensions (physical function, role physic, pain and health perception) and the general physical function of Short Form 36

	Raw Score	NYHA
Fatigue scale	0.36	0.42
Total physical activity (mets)	-0.02	-0.02
General physical health (SF36)	-0.40	-0.40

Physical function	-0.33	-0.36
Role physical	-0.27	-0.30
Bodily pain	-0.35	-0.38
General health perception	-0.47	-0.44

### Concurrent calibration

**Figure 1:**
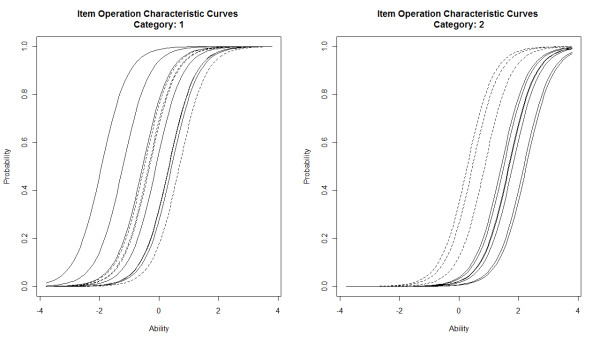
**Item operation characteristic curves^1 ^for 4 anchor items (dashed lines) and 7 observers for NYHA classification (solid lines)**. ^1^Item operation characteristic curves (IOCC) for category k represent the probability of endorsing categories higher than k conditional on subject's ability.

Inspection of the thresholds between classes I-II and II-III provided information about the ability extremes (Table 5). Estimates for the first threshold ranged from -1.92 to 0.69 (median = -0.10) standard deviations of ability, and for the second threshold ranged from 0.27 to 2.26 (median = 1.69). Observers 4 (t1 = -1.92) and 7 (t1 = 0.43) showed the lowest and highest first threshold, respectively, while for the second threshold it was observer 4 (t2 = 1.24) and 8 (t2 = 2.26), respectively (Figure 1). "Effort" was the anchor item whose first threshold was closest to the median of the observers' first threshold, that is, this anchor item is considered to distinguish classes I and II. The "effort" anchor item second threshold was closest to the median of the observers' second thresholds, that is, these anchor items are considered to distinguish classes II and III.

**Table 5:** One-dimensional 2 parameter logistic graded response model with equal discrimination parameters across items

	Threshold 1^1^ t_1 _(se)	Threshold 2^2^ t_2 _(se)	Item Discrimination^3^ Β (se)
"Dyspnea"	0.688 (0.106)	---	2.268 (0.176)
"Distance"	-0.349 (0.104)	0.278 (0.094)	2.268 (0.176)
"Stairs"	-0.493 (0.102)	0.459 (0.390)	2.268 (0.176)
"Effort"	-0.316 (0.099)	0.865 (0.123)	2.268 (0.176)
Observer 1	-0.549 (0.283)	1.503 (0.287)	2.268 (0.176)
Observer 2	-0.099 (0.168)	1.692 (0.631)	2.268 (0.176)
Observer 3	---	---	---
Observer 4	-1.920 (0.253)	1.420 (1.237)	2.268 (0.176)
Observer 5	0.331 (0.312)	1.671 (0.309)	2.268 (0.176)
Observer 6	-1.211 (0.449)	2.178 (0.728)	2.268 (0.176)
Observer 7	0.430 (0.269)	1.804 (3.418)	2.268 (0.176)
Observer 8	0.339 (0.247)	2.263 (0.305)	2.268 (0.176)

^1^Threshold 1 - level of ability above which 50% of subjects were NYHA class II-III

^2^Threshold 2 - level of ability above which 50% of subjects were NYHA class III

^3^Item discrimination - represents the slope of the item characteristic curves at the value of the threshold and indicates the extent to which the item is related to the ability

se: standard error.

The results of the calibration with the 1-PL graded response model showed that the observers and the patient anchor items showed a high discrimination (β = 2.27, standard error = 0.176).

### Quality of the calibration

The agreement between the NYHA classification according to the thresholds estimated for each observer for the ability and the observers' NYHA classification observed (target items) ranged from 76 to 89% with a median of 88%, the weighted Kappa ranged from 0.42 to 0.83 with median of 0.61 (table 6). This means that after taking into account the discrepancies in thresholds between observers, their NYHA classification is well predicted by the ability, with a substantial agreement.

**Table 6:** Agreement between the observers and between the observers and the ability estimated by the concurrent calibration

	Observer 1	Observer 2	Observer 4	Observer 5	Observer 6	Observer 7	Observer 8	Ability
**Observer 1**	---	82.89^1^	70.72^1^	65.40^1^	72.24^1^	59.32^1^	62.36^1^	0.76^1^
**Observer 2**	0.68^2^	---	53.61^1^	82.51^1^	58.17^1^	76.43^1^	79.47^1^	0.88^1^
**Observer 4**	0.20^2^	0.09^2^	----	36.12^1^	90.87^1^	30.04^1^	33.08^1^	0.87^1^
**Observer 5**	0.43^2^	0.69^2^	0.06^2^	---	40.68^1^	93.92^1^	96.96^1^	0.88^1^
**Observer 6**	0.21^2^	0.12^2^	0.00^2^	0.06^2^	---	38.4^1^	42.21^1^	0.89^1^
**Observer 7**	0.33^2^	0.57^2^	0.01^2^	0.87^2^	0.05^2^	---	96.2^1^	0.88^1^
**Observer 8**	0.36^2^	0.62^2^	0.00^2^	0.94^2^	0.06^2^	0.92^2^	---	0.79^1^
**Ability**	0.56^2^	0.80^2^	0.42^2^	0.83^2^	0.47^2^	0.77^2^	0.61^2^	---

^1^Upper triangle shows the % of absolute agreement

^2^Lower triangle shows the weighted kappa.

The agreement between observers predicted classifications for all individuals according to the thresholds estimated for each observer for the ability ranged from 30 to 97% with a median of 65%, the weighted Kappa ranged from 0.00 to 0.94 with median of 0.21. This means that without taking into account the discrepancies in thresholds between observers, the agreement between NYHA observers classification is fair.

## Discussion

Several studies have shown that the NYHA classification is valid but not reproducible [2,4], and associated with symptom burden, quality of life, exercise capacity, and increased risk of ischemic stroke [18-20]. Nevertheless, the NYHA classification was originally designed as a clinical, not a research tool. Although much has been written regarding the limitations of the NYHA of classification as an outcome measure [21], investigators continue to use it in clinical research. The popularity of the NYHA classification system is based on its simplicity [4]. Any system that might replace it should be more accurate without being more complex. So the aim of this study was not to build a new system but to improve the NYHA system. To do so, we used IRT models to equate and calibrate a large number of observers on the same scale; by doing so, we were able to identify observers with lower and higher thresholds for classification, as well as to understand the relations with anchor items across the ability continuum, and to improve the NYHA classification system.

The present study objectively indicates the main reason why several studies have reported low inter-observer reliability and, consequently, the limited usefulness of the NYHA classification as an outcome measure. The main reason is the existence of discrepant thresholds between observers in the definition of NYHA class I, II and III individuals. Although the observers in study were experienced physicians well trained in the management of heart failure, there were still discrepancies between their (subjective) evaluations.

The focus should therefore be on the identification of differences between the evaluations of the observers and on the calibration of those classifications.

Although intra-observer reliability is more important to interpret changes in NYHA class in the individual patient who is assessed repeatedly by the same physician, inter-observer variability is of special concern when patients are assessed by different physicians. This is particularly important, in practice, in unscheduled visits to the clinic or the emergency department, where patients are not assessed by their usual attendant. These unscheduled visits are usually due to worsening symptoms and an increase in NYHA class, in comparison with the previous clinical state, is used as a criterion for clinical decisions such as hospital admission and intensity of therapy adjustment such as use of intravenous medication.

Therefore, in each setting the NYHA classification is to be used, it would be useful to identify the differences between the assessments of the observers and calibrate their classifications. For the calibration with the IRT methodology to be possible, a set of anchor items is needed. These items should be reliable and valid. In this sample, the 4 anchor items combined with each target item showed good homogeneity (strong first factor) and reliability (alpha > 0.61). Furthermore, these items showed content validity on the basis of a previous study [3], which concluded that the self-reported distance (70%) and difficulty in climbing stairs (60%) were the items more commonly used by senior cardiologists and trainees in cardiology to classify patients in NYHA classes. Our study showed that these anchor items had a strong association with the NYHA classification and that had a similar association with scales that measure related constructs. So these results confirm the reliability of the anchor items and their validity to assess the same construct as the NYHA classification.

The improvement in the absolute agreement (65% to 88%) between the ability scale predictions of the NYHA classification between observers and the ability scale predictions of the NYHA classification with the observers' NYHA classifications observed, show how the subjectivity of the thresholds can affect the reliability of the NYHA classification. At the same time this improvement confirms the quality of the calibration obtained.

The calibration methodology can be useful to improve the reliability between observers in clinical practice and research settings. In clinical practice it is possible to use the anchor items' relations with ability to explain the differences between observers and give guidelines to improve the inter-observers reliability. For example, if we wanted to calibrate the threshold between NYHA I and II for all observers, we would advise all observers to use endorsement of the second category of the "Effort" item for the definition of class NYHA II. Similarly if we wanted to calibrate the threshold between NYHA II and III we would advise all observers to use endorsement of at least the third category of the "effort" item. In research settings the ability scale, defined using both the anchor items and an operator's classification, can be used as a refined NYHA classification, independently of the subjectivity of the observers.

The major limitation of this study is its small size. Whereas the minimum number of individuals required to properly fit a 1-PL model is 200 [22], only slightly less than the 263 individuals assessed here, a proper 2-parameter logistic (2-PL) GRM allowing the slope to vary among the items would require a larger sample size. An inadequate sample size would be expected to yield unstable item parameters and higher standard errors, which was the case in our study.

In the present study, each individual was assessed by only one observer, opposed to the ideal situation where that individual would be assessed by all observers. We do not think of this as a limitation. When we compared the individuals assessed by each of the observers there were no statistically significant differences in sex, clinical history, systolic blood pressure, education and left ventricular systolic dysfunction; only age, body mass index and diastolic blood pressure showed small differences. Consequently, overall the individuals that each observer assessed were very similar. On the other hand, the anchor items were related to each observer's NYHA classification. So even if the sample assessed by each observer was very discrepant, the anchor items would guarantee a good calibration. Therefore we are confident that this limitation did not have a major impact on the results.

The anchor items proposed to calibrate the NYHA classifications are not assumed to be the gold standard and are not intended replace the NYHA classification by themselves. The study only validated these anchor items against the NYHA classifications, supporting that they could be used to calibrate different observers in using NYHA classification. We do not intend to question the validity of either the anchor items or NYHA classification to measure true functional capacity, in which case we would need to confront each of them with quantitative measures of functional capacity like the 6-minute walk test or a cardiopulmonary exercise test with measurement of oxygen consumption.

Self-reported distance is a subjective measure and many factors influence a patient's answer, including psychosocial factors and perceptions of distance. Patients' ability to estimate 100 m, 500 m and 2500 m distance was shown to be poor [3]. However, the use of additional anchor items is expected to attenuate the impact of this potential error in each of them.

The physicians were aware of patients' responses to the 4-anchor items. It is therefore possible that this fact influenced their ratings and thus violated the assumption of local independence of the statistical model. Separate calibration with the mean/mean method [23] was use as sensitivity analysis (data not shown) and the results obtained were similar to the concurrent analysis, also there were no significant differences between the observed and expected frequencies of items for the 7 observers models and only one pair of anchor items in 1 out of the 7 observers graded response model (observer 2) showed local dependencies.

The generalisation of the calibration method proposed is limited by the lack of individuals classified as NHYA class IV.

## Conclusions

In conclusion, this study showed that the thresholds of the NYHA classification between observers were very discrepant and that concurrent calibration through IRT models can be used to calibrate a large number of observers on the same scale. It provides a way to minimize the reliability problem of NYHA classification. This type of approach can be useful to minimize the inter-observer variability in other classifications based on patient's and/or physicians's perception.

## Competing interests

The authors declare that they have no competing interests.

## Authors' contributions

MS participated in the study design, performed the statistical analysis and helped to draft the manuscript. RG performed the statistical analysis and helped to draft the manuscript. PL, MA, PB and AA participated in the study design and helped to draft the manuscript.

All authors read and approved the final manuscript
